# Transcriptional regulatory networks controlling woolliness in peach in response to preharvest gibberellin application and cold storage

**DOI:** 10.1186/s12870-015-0659-2

**Published:** 2015-11-18

**Authors:** Camila Pegoraro, Alice Tadiello, César L. Girardi, Fábio C. Chaves, Vera Quecini, Antonio Costa de Oliveira, Livio Trainotti, Cesar Valmor Rombaldi

**Affiliations:** Plant Genomics and Breeding Center, Universidade Federal de Pelotas, Campus UFPel Capão do Leão, Pelotas, RS 96010-900 Brazil; Current Address: Embrapa Uva e Vinho, Rua Livramento 515, Bento Gonçalves, RS 95700-000 Brazil; Department of Biology, University of Padova, Viale G. Colombo, Padova, 3, 35121 Italy; Current Address: Research and Innovation Centre, Fondazione Edmund Mach, Via Mach 1, San Michele all’Adige, Trento, 38010 Italy; Embrapa Uva e Vinho, Rua Livramento 515, Bento Gonçalves, RS 95700-000 Brazil; Departament of Food Science and Technology, Universidade Federal de Pelotas, Campus UFPel Capão do Leão, Pelotas, RS 96010-900 Brazil

**Keywords:** Chilling injury, Cold storage, Genome wide gene expression, Gibberellic acid, *Prunus persica*

## Abstract

**Background:**

Postharvest fruit conservation relies on low temperatures and manipulations of hormone metabolism to maintain sensory properties. Peaches are susceptible to chilling injuries, such as ‘woolliness’ that is caused by juice loss leading to a ‘wooly’ fruit texture. Application of gibberellic acid at the initial stages of pit hardening impairs woolliness incidence, however the mechanisms controlling the response remain unknown. We have employed genome wide transcriptional profiling to investigate the effects of gibberellic acid application and cold storage on harvested peaches.

**Results:**

Approximately half of the investigated genes exhibited significant differential expression in response to the treatments. Cellular and developmental process gene ontologies were overrepresented among the differentially regulated genes, whereas sequences in cell death and immune response categories were underrepresented. Gene set enrichment demonstrated a predominant role of cold storage in repressing the transcription of genes associated to cell wall metabolism. In contrast, genes involved in hormone responses exhibited a more complex transcriptional response, indicating an extensive network of crosstalk between hormone signaling and low temperatures. Time course transcriptional analyses demonstrate the large contribution of gene expression regulation on the biochemical changes leading to woolliness in peach.

**Conclusion:**

Overall, our results provide insights on the mechanisms controlling the complex phenotypes associated to postharvest textural changes in peach and suggest that hormone mediated reprogramming previous to pit hardening affects the onset of chilling injuries.

**Electronic supplementary material:**

The online version of this article (doi:10.1186/s12870-015-0659-2) contains supplementary material, which is available to authorized users.

## Background

Typically, the shelf life of peach fruit (*Prunus persica* L. Batsch.) is short due to its fragility, fast loss of pulp firmness and decay susceptibility. Thus, postharvest conservation is based on methods that decrease pathogen inocula, and reduce fruit metabolism [1]. Cold storage (CS) has been the main method used to increase peach shelf life. However, a wide range of factors, such as cultivar, ripening stage at harvest, temperature and time of storage, contribute to the occurrence of physiological disorders, commonly known as chilling injury, including woolliness or loss of juice, pit darkening and reddening, flesh breakdown and others [2, 3].

In peaches, woolliness occurrence is frequent after long-term CS, even when the fruits are kept at temperatures as low as 0 °C [4–7]. Chilling injuries are complex phenotypes, likely to be controlled by a wide range of genetic, developmental, anatomical and physiological factors [2, 3, 8, 9]. Exogenous application of GA to peach and nectarine fruits on the tree at the initial stages of pit hardening has been demonstrated to effectively reduce woolliness incidence [3, 5, 6]. Preharvest hormone application has been demonstrated to reduce chilling injuries a reduced number of cultivars, such as Chimarrita and Chiripá [3, 5, 6]. The responses induced by the hormone that lead to the reduced occurrence of the woolly phenotype remain largely unknown. Exogenous GA application in citrus has been demonstrated to induce pleiotropic and previously unreported effects [10].

Pectin metabolism is currently considered one important effector of woolliness in peach [2], although the molecular mechanisms involved in its onset remain elusive. Reduced accumulation of transcripts from genes related to normal ripening processes, such as those involved in ethylene biosynthesis and signaling, cellular respiration, volatile compounds biosynthesis, endocellular transport, protein folding, lipid turnover, cell wall disassembling and redox system, has been correlated to chilling disorders [11–18]. The abnormal ripening processes under cold storage are thought to trigger wooliness at the transcriptional level. Targeted proteomic approaches have also demonstrated similar behavior of protein and enzyme activity levels [1, 5].

In the current work, we have taken advantage of the GA-responsive genomic context, consisting of the cultivar Chimarrita, to investigate the large-scale transcriptional profile of peaches, subjected to hormone application at the initial stages of pit hardening, at harvest and after chilling injury inducing storage conditions. In addition, time course analyses of the woolly phenotype development and candidate gene expression were performed on GA-treated and control fruits submitted to CS, before the onset of the disturbance. Taken together, our results demonstrate that a complex interplay between transcriptional programs controlled by GA and low temperatures underlies cellular and developmental mechanisms associated to woolliness in peaches.

## Results

### Application of GA at pit hardening stage reduces woolliness

**Fig. 1:**
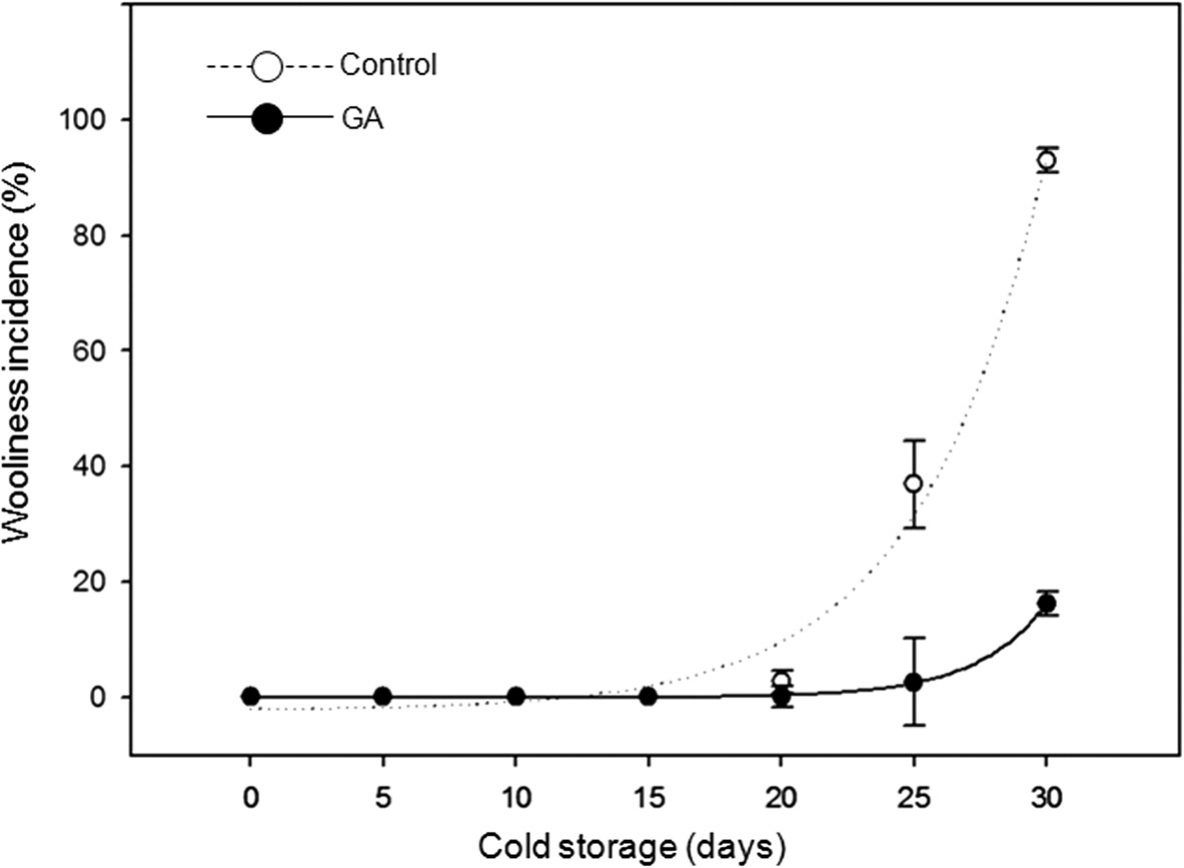
Woolliness symptom occurrence in preharvest GA untreated (Control) and GA treated peaches (GA). Fruits were stored for 30 days under cold storage (CS – 0 ± 0.5 ° C and 90 ± 5 % of relative humidity) and ripened at room temperature (RT - 25 ± 2 ° C) for 2 days. The GA treatment was carried out in the pre harvest before pit hardening of peaches

The incidence of woolliness remained undetected for control and GA treated fruits up to 15 days under CS (Fig. 1). After this period, the frequency of woolly fruits steeply increased in untreated control peaches (Fig. 1). Preharvest application of exogenous GA significantly prevented the incidence of woolliness after CS (Fig. 1). After 30 days under CS and two days at RT, approximately 100 % of the control fruits exhibited the physiological disorder (Fig. 1). In contrast, the incidence of woolliness in fruits harvested from trees sprayed with GA at the onset of pit hardening was more than six fold smaller (16 %) (Fig. 1).

### Genome wide transcriptional profiling of peach under postharvest conditions

**Fig. 2:**
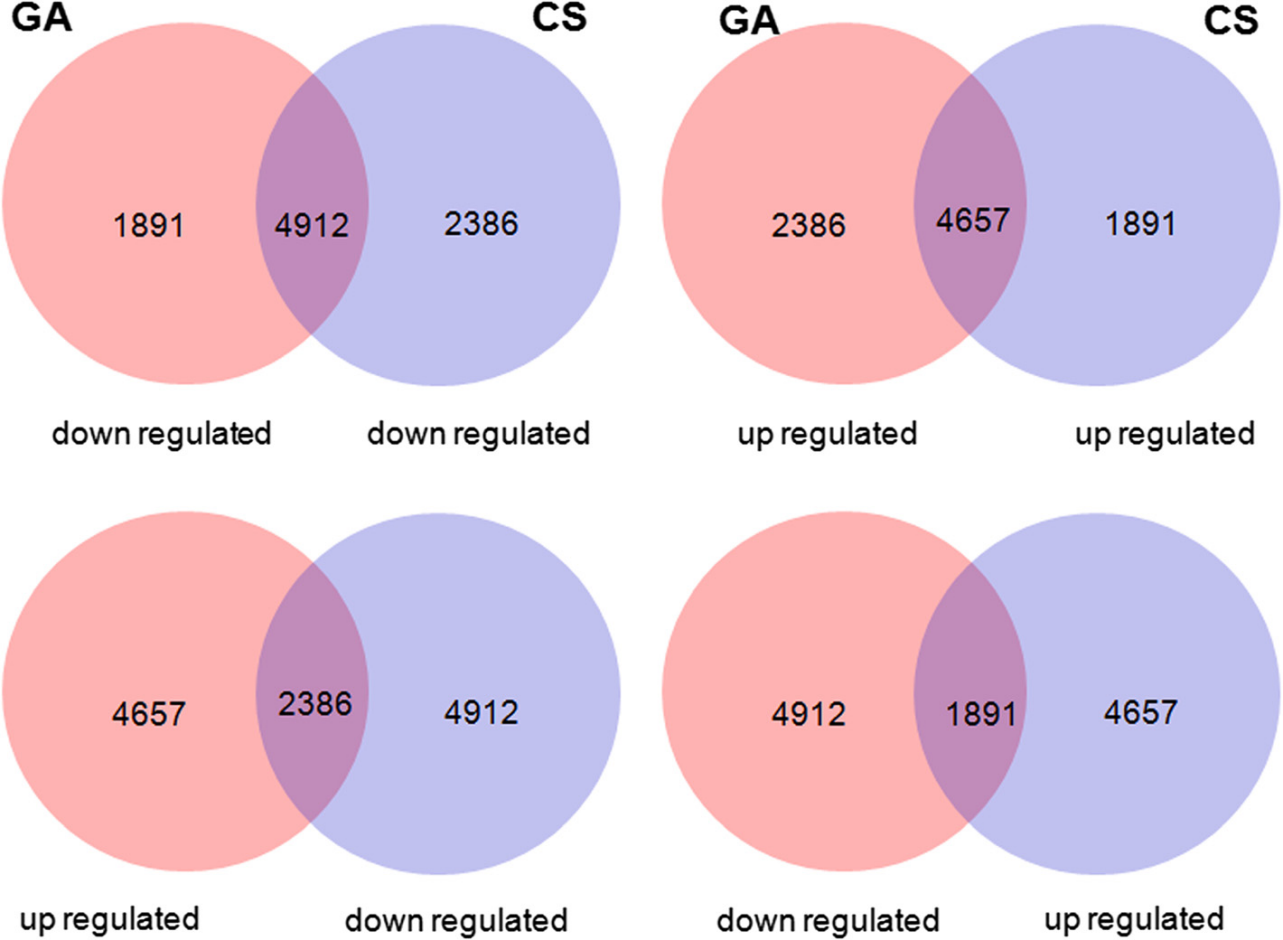
Differentially regulated genes in peaches submitted to GA and CS factors. GA: gibberellic acid; CS: cold storage. The treatments used for determination of GA effect were C (harvest) and CS - level GA 0 – against GA and GACS – level GA 50 mgL^−1^. The treatments used for determination of CS effect were C and GA – level CS 0 – against CS and GACS – level CS 0 °C. Differentially expressed genes were identified by LIMMA analysis and classified as up or down regulated by log_2_ fold change

Genome wide expression analyses, employing 28,689 protein-coding transcripts from the peach transcriptome were performed for control and GA treated fruit at harvest and after CS. Factorial analysis of the expression data revealed extensive transcriptional changes in response to CS and GA, with 48.26 % (13846) of the genes being differentially expressed (Fig. 2, Additional file 1: Table S1). From overlap analysis, approximately 34 % of the genes exhibiting differential expression in response to the tested factors (GA, CS) were commonly down (35.4 %, 4912) and (33.63 %, 4657) up regulated (Fig. 2), although induction and/or repression levels were significantly different between GA and CS. Gene sets exhibiting exclusive regulation by CS or GA were also detected (Fig. 2). A subset of transcripts exhibit opposite responses to GA and CS treatments, being 17.23 % (2386) induced by GA and repressed by CS (Fig. 2) and 13.7 % (1891) repressed by GA and induced by CS (Fig. 2).

**Fig. 3:**
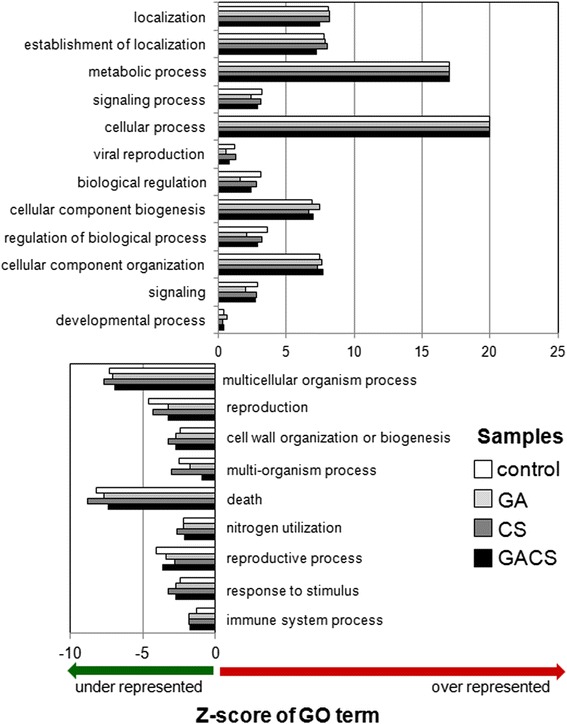
Singular enrichment analysis of GO biological process categories for differentially expressed genes. GO classifications of the responsive genes were compared to those from the peach genome and bar length represents z-score for the GO in each treatment. The treatments correspond to control (untreated fruits at harvest), GA (GA treated fruits at harvest), CS (untreated fruits submitted to cold storage) and GACS (GA treated fruits submitted to cold storage)

**Fig. 4:**
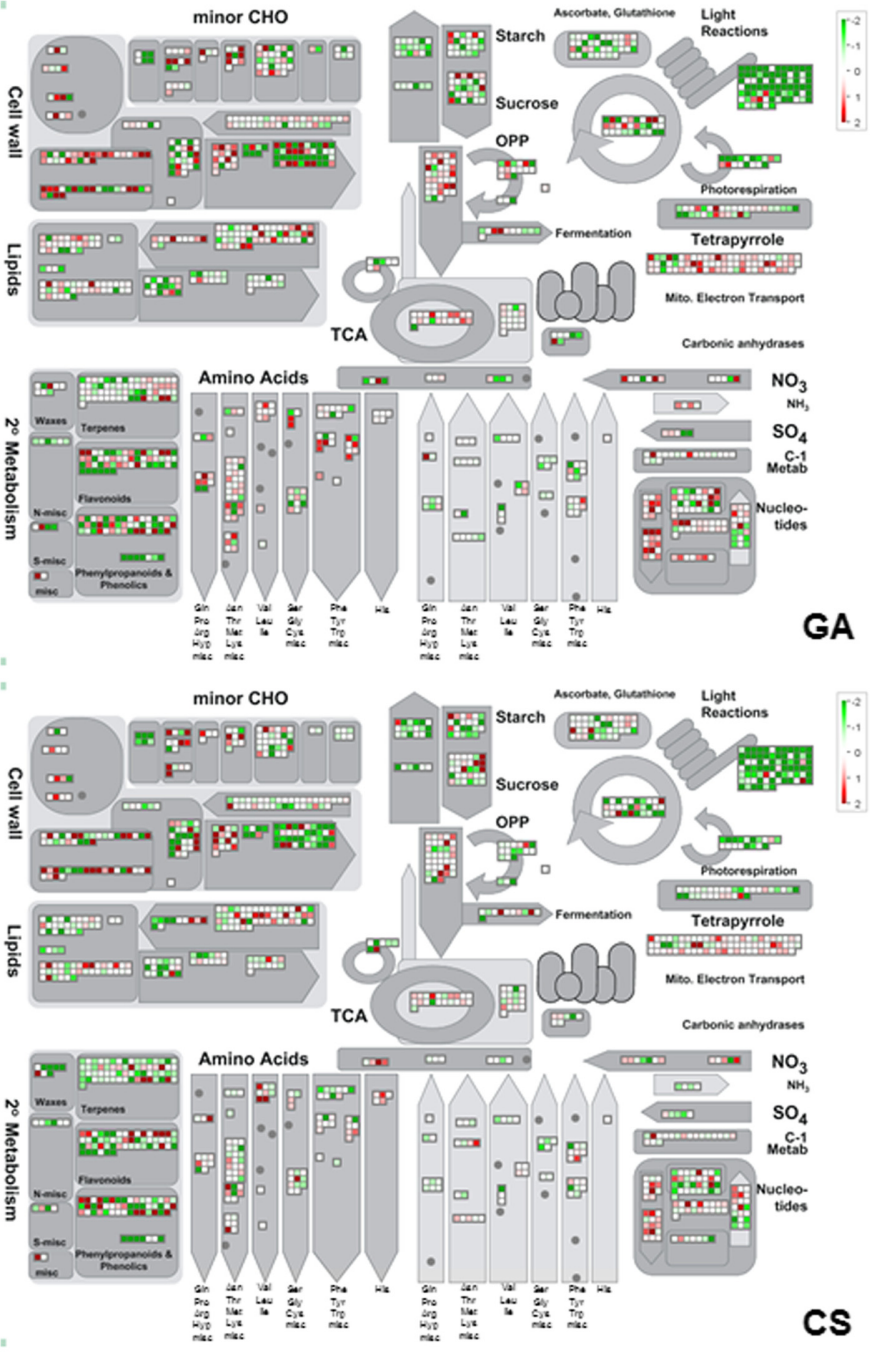
General metabolism classification of differentially expressed genes in response to GA and CS. The treatments used for determination of GA effect were C (harvest) and CS - level GA 0 – against GA and GACS – level GA 50 mgL^−1^. The treatments used for determination of CS effect were C and GA – level CS 0 – against CS and GACS – level CS 0 °C. Log_2_ fold changes are represented as color scale for GA x no GA (**a**) and cold x no cold (**b**) contrasts. Positive values (red) correspond up regulated genes and negative values (green), to down regulated genes

Differentially regulated genes classified to gene ontology (GO) classes of cellular, metabolic and developmental processes were significantly overrepresented in comparison to reference peach transcriptome (Table 1, Fig. 3). In contrast, GO classes corresponding to cell death and immune system responses were underrepresented in all investigated conditions (Table 1, Fig. 3). Metabolic classification of the genes exhibiting significant responses to GA and CS according to the MapMan scheme revealed transcriptional shifts associated to primary and specialized metabolism (Fig. 4), with a predominantly repressive effect of CS on global gene expression. CS and GA treatment significantly affected the expression of the coding sequences for a wide range of cell wall metabolism associated proteins, such as cellulose syntases, pectinesterases, polygalacturonases, pectate lyases, xyloglucan endotransglycosyilases and expansins (Fig. 4). Similarly, genes associated to carbohydrate, lipid, specialized metabolism, amino acids, nucleotides, fermentation, tetrapyrrole and photorespiration (Fig. 4) were distinctly regulated in response to low temperatures and GA treatment.

**Table 1:** Parametric analysis of gene set enrichment of GO terms in response to GA and CS

		Number				
GO class	Description	Differentially expressed (13870)	Reference genome (28702)	Enrichment fold	*p*-value	FDR^a^
Biological process						
GO:0006412	translation	323	396	2.268725143	4.30E-27	1.50E-23
GO:0009987	cellular process	2356	5297	1.237142882	1.30E-21	2.40E-18
GO:0044237	cellular metabolic process	1887	4133	1.269933684	1.40E-20	1.70E-17
GO:0008152	metabolic process	2703	6308	1.191870055	3.20E-18	2.80E-15
GO:0044267	cellular protein metabolic process	890	1791	1.382193889	5.60E-16	3.90E-13
GO:0009058	biosynthetic process	1038	2202	1.311156664	7.80E-14	4.60E-11
GO:0044249	cellular biosynthetic process	968	2075	1.297573011	3.80E-12	1.90E-09
GO:0044281	small molecule metabolic process	317	558	1.580154713	8.10E-11	3.60E-08
GO:0010467	gene expression	747	1589	1.307588981	4.80E-10	1.80E-07
GO:0019538	protein metabolic process	1022	2273	1.250621837	5.10E-10	1.80E-07
Molecular function						
GO:0003735	structural constituent of ribosome	234	265	2.456091438	5.60E-23	1.20E-19
GO:0005198	structural molecule activity	252	303	2.313302674	3.30E-22	3.60E-19
GO:0003824	catalytic activity	2579	6355	1.128782675	2.30E-09	1.70E-06
GO:0016874	ligase activity	146	246	1.650791775	1.60E-06	0.00087
GO:0019001	guanyl nucleotide binding	121	212	1.587537735	4.30E-05	0.017
GO:0032561	guanyl ribonucleotide binding	119	209	1.58370841	5.40E-05	0.017
GO:0005525	GTP binding	119	209	1.58370841	5.40E-05	0.017
GO:0016879	ligase activity, forming carbon-nitrogen bonds	100	171	1.626591271	9.70E-05	0.026
Cellular component						
GO:0005622	intracellular	1106	2064	1.490458821	4.00E-28	3.00E-25
GO:0005737	cytoplasm	496	727	1.8976749	8.70E-28	3.30E-25
GO:0044444	cytoplasmic part	411	570	2.005587037	1.90E-26	4.70E-24
GO:0032991	macromolecular complex	469	694	1.87969731	1.10E-25	2.00E-23
GO:0030529	ribonucleoprotein complex	259	294	2.450343564	3.50E-25	5.20E-23
GO:0044424	intracellular part	852	1555	1.523995726	1.00E-23	1.30E-21
GO:0005840	ribosome	234	265	2.456091438	5.60E-23	6.00E-21
GO:0044464	cell part	1729	3696	1.301180597	4.30E-22	3.60E-20
GO:0005623	cell	1729	3696	1.301180597	4.30E-22	3.60E-20
GO:0043229	intracellular organelle	657	1185	1.542132063	2.80E-19	1.90E-17

^a^ *FDR* false discovery rate was estimated by Benjamini–Hochberg–Yekutieli procedure at AgriGO (http://bioinfo.cau.edu.cn/agriGO/index.php)

A large number of genes associated to photosynthesis light reactions were differentially regulated in peach fruit in response to CS and GA application (Fig. 4). The application of GA and storage of fruit under CS promoted the differential expression of sequences coding for light harvesting complexes, photosystem II reaction centers, photosystem II core complexes, photosystem I subunits and photosystem II subunits (Fig. 4).

### Gene set enrichment analyses (GSEA) of woolliness associated candidate probes

**Fig. 5:**
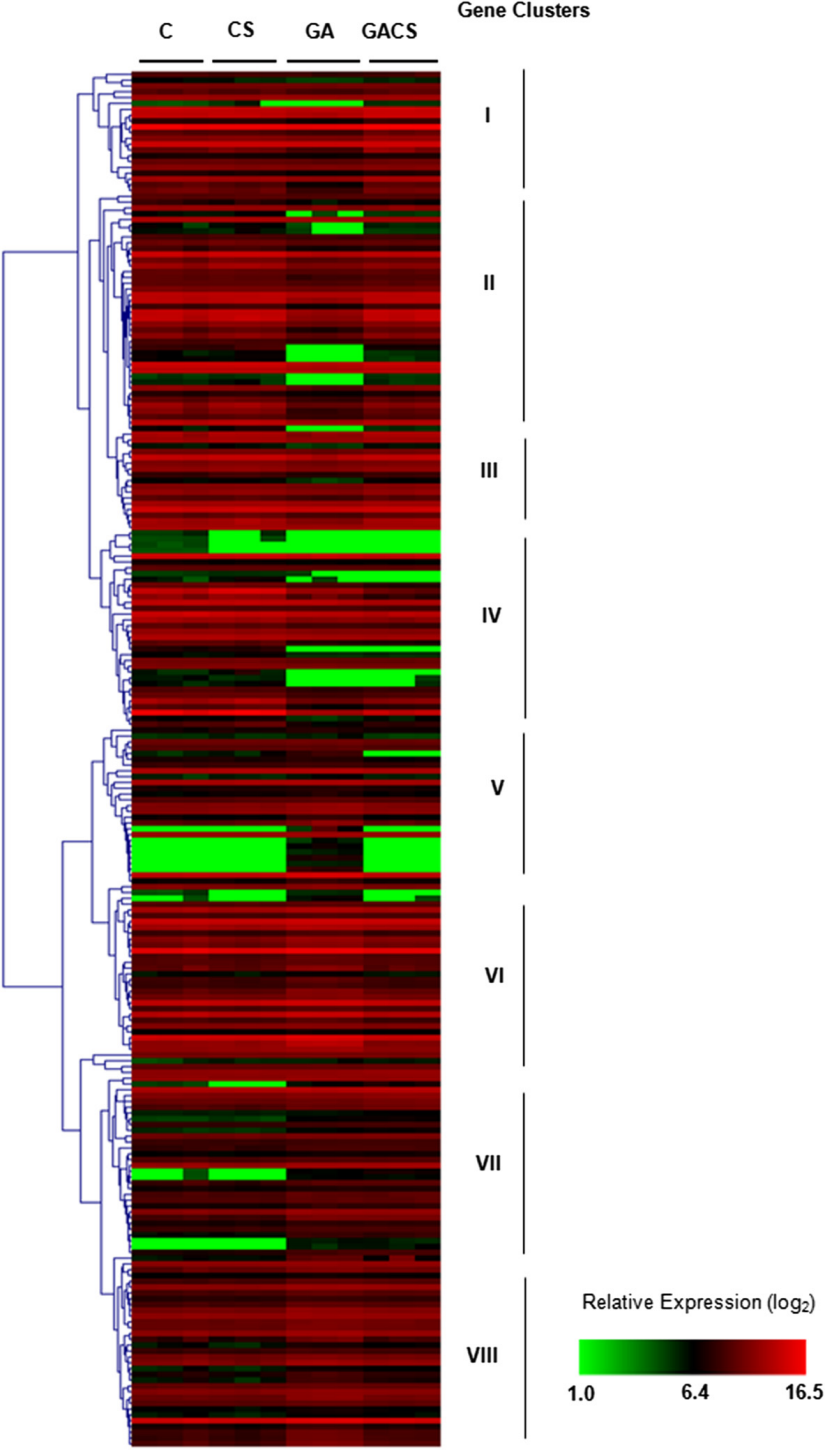
Enrichment analysis of differentially expressed genes associated to cell wall metabolism in peach. Experimental conditions correspond to C (control fruits, 2 days at RT); CS (hormone untreated fruits, under cold storage for 15 days); GA (hormone treated fruits, 2 days at RT) and GACS (hormone treated fruits, under cold storage for 15 days). Roman numbers represent expression clusters, by Hierarchical Clustering using Pearson Correlation

To gain insight in the processes associated to the onset of woolliness, we have investigated the transcriptional behavior of genes known to be involved in cell wall metabolism and hormonal regulation by gene set enrichment analyses (GSEA). Eight distinct expression patterns were identified by clustering analyses of the transcriptional behavior of cell wall metabolism genes in peach in response to CS and GA. Exogenous GA application repressed the expression of a subset of genes in the first two clusters. A third subset of cell wall associated genes, which includes expansin and pectinesterase coding sequences, exhibited repressed transcription equally mediated by CS and GA (Fig. 5). The transcriptional profile of a large number of genes involved in cell wall processes in clusters IV, V and VI, remained virtually unchanged in response to both investigated factors (Fig. 5). In contrast, CS relieved the transcriptional repression of a subset of genes associated to cell wall metabolism in peach (cluster VII and VIII), including those related to carbohydrate metabolism and endomembrane transport (Fig. 5).

**Fig. 6:**
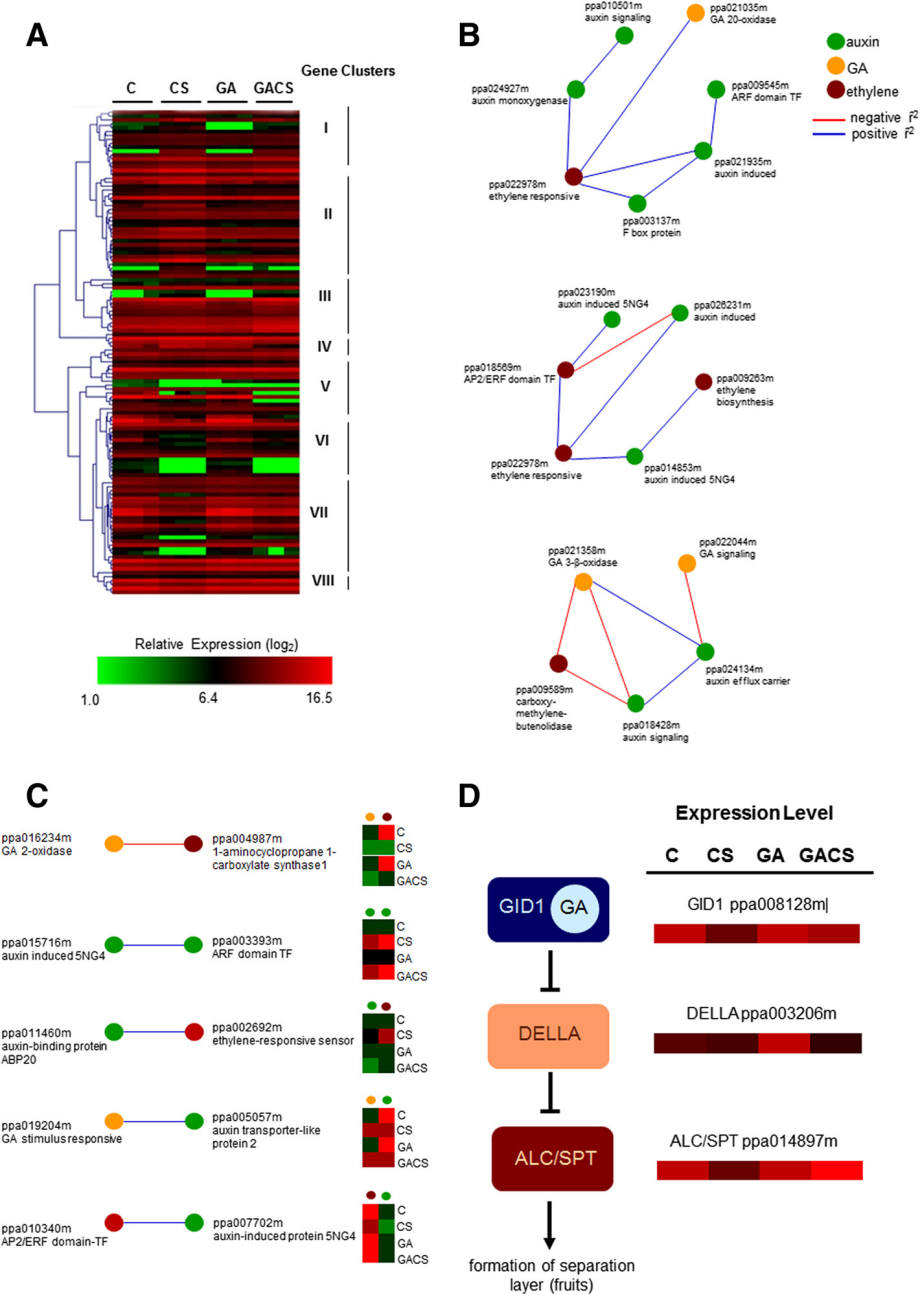
**a** Enrichment analysis of differentially expressed genes associated to hormone metabolism in peach. Experimental conditions correspond to C (control fruits, 2 days at RT); CS (hormone untreated fruits, under cold storage for 15 days); GA (hormone treated fruits, 2 days at RT) and GACS (hormone treated fruits, under cold storage for 15 days). Roman numbers represent expression clusters, by Hierarchical Clustering using Pearson Correlation. **b, c** Relevance networks constructed from the expression pattern of genes associated to hormone metabolism. Associations with negative ȓ^2^ are represented in red and those with positive ȓ^2^, in blue. Probes with no associations at 0.80 were removed. **d** Expression of GA signaling components in endocarp layer separation in response to GA and CS

Genes associated to hormonal responses exhibit a more complex response pattern to GA exogenous application and CS in peaches (Fig. 6). A subset of transcripts corresponding to gibberellin biosynthesis oxidases and ethylene responsive transcription factors (AP2/ERF) (cluster I) was induced by CS, although subjected to an antagonistic effect of GA application (Fig. 6). A similar profile, although less marked, was observed for genes coding for auxin signaling partners and biosynthesis enzymes (cluster II and III). The combined action of cold storage and gibberellic acid treatment led to the repression of a small set of genes associated to hormone responses in cluster IV, with the majority of them belonging to the auxin, ethylene and gibberellin metabolism. In contrast, the transcription of other groups of hormone-related genes was repressed by CS with an inductive effect of GA (clusters V, VI and VII) (Fig. 6). Significant differential regulation was also observed for peach homologs of GA receptor *GID*1, one sequence coding for a DELLA repressor and *ALCATRAZ*/*SPATULA* transcription factor in response to CS and GA (Fig. 6). A small number of hormone associated genes were shown to respond individually to a single factor, with the vast majority of the sequences exhibiting transcriptional changes in response to both factors (Fig. 6). Corregulated modules consisting of GA, auxin and ethylene metabolism were evidenced by relevance network analysis, including a module of genes coding for auxin biosynthesis and signaling, GA catabolism and an ethylene responsive protein, auxin mediated signaling and ethylene responses and biosynthesis (Fig. 6). The opposite transcriptional behavior of the genes coding for GA and ethylene biosynthesis key enzymes in response to GA and CS was also shown (Fig. 6). In contrast, the transcription of sequences coding for auxin, GA and ethylene signaling partners exhibited a similar response to the investigated factors (Fig. 6).

### Reverse transcription quantitative PCR gene expression analyses

Microarray data validation and time course expression analyses of candidate genes associated to woolliness under CS were performed by RT-qPCR. The expression patterns observed by microarray analyses were consistently confirmed by RT-qPCR for genes associated to cell wall metabolism, redox system and photosynthesis (Additional file 2: Figure S1). The expression pattern of approximately 70 % of the investigated genes was similar between the microarray and RT-qPCR.

**Fig. 7:**
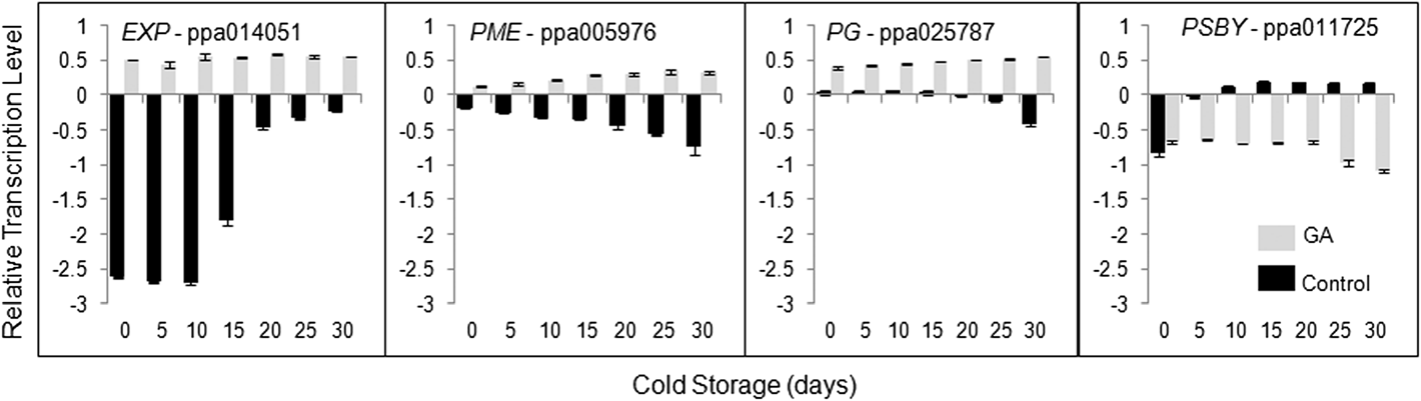
Expression kinetics of cell wall metabolism and photosynthesis genes during CS and GA-treated peaches. Values correspond to the mean ± SD (*n* = 3)

Time course analyses of cell wall metabolism genes during CS have demonstrated that the transcriptional profile of three genes associated to cell wall metabolism and one gene associated to photosynthesis were discrepant between control and GA treated fruit (Fig. 7). Interestingly, the most striking transcriptional differences between hormone treated and control fruit were found at the first half of the low temperature period for an *EXPANSIN* (*EXP*, ppa014051) coding sequence. In contrast, for *PECTIN METHYL ESTERASE* (*PME*, ppa005976) and *POLYGALACTURONASE* (*PG*, ppa025787m), the differences were greater at later stages of CS (Fig. 7). The transcription of a gene coding for a *PHOTOSYSTEM II CORE COMPLEX PROTEIN* (*PSBY*, ppa011725m) was repressed in GA treated fruit submitted to CS (Fig. 7).

## Discussion

### Effect of GA application on peach fruit

The role of GA in fruit ripening and development remains largely unknown, although the effect of endogenous or exogenous GA on fruit growth has been shown for several species [19, 20]. Previously, we have demonstrated that exogenous application of GA at the initial stages of pit hardening effectively reduces the incidence of chilling injuries in responsive cultivars [5, 6]. Applications at later stages did not significantly affect the frequency of the disorder in stored fruit [5, 6]. Thus, the processes leading to reduced frequencies of woolliness in response to GA application are confined to pre lignification of the endocarp. In dry, dehiscent fruit of model plant *Arabidopsis thaliana*, GA has been demonstrated to negatively regulate, via DELLA repressors, the bHLH transcription factor ALCATRAZ (ALC), involved in the determination of the cell fate for the non-lignified valve margin tissues promoting fruit dehiscence [21, 22]. ALC function is partially redundant to that of another bHLH transcription factor, SPATULA (SPT) [23]. The transcriptional regulator INDEHISCENT (IND) activates the transcription of a GA-activating enzyme (GAOX1); thus, resulting in accumulation of the hormone in the separation valve layers leading to the dissociation of the DELLA repressor from ALC, allowing cell fate specification [22]. Recently, the essential role of gibberellin-mediated signaling components in fruit development has been demonstrated for plum (*Prunus salicina* L.) [20].

Peach is a fleshy fruit with hardened endocarp, termed drupe. In peach genome clear homologs of *ALC* and *IND* are absent and the most similar genes identified exhibit a non specific endocarp transcription pattern [24, 25]. In contrast, the expression of peach *SPT* homolog is consistent with a role in cell fate determination in endocarp margins [26]. Thus, in peach, *SPT* is likely to be an important factor controlling the determination of endocarp margins. Transcription of homologs of GA receptor *GID*1 and signaling components, including DELLA repressors and SPT transcription factor, was significantly altered by exogenous GA application and CS (Fig. 6). Interestingly, while all three components were repressed in response to CS and induced by GA application, the combination of both factors led to the induction of *GID*1 and *SPT* transcription (Fig. 6). The responsive DELLA coding sequence was down regulated by the combination of exogenous GA application and cold temperatures (Fig. 6). In other plant species the application of bioactive forms of gibberellin have been shown to stimulate the degradation of the repressive DELLA proteins and result in the loss of stress tolerance [27, 28]. Several corregulated modules consisting of genes associated to auxin, GA and ethylene metabolism were demonstrated by relevance network analyses of functionally annotated sequences (Fig. 6). Interestingly, exogenous GA application restored the expression levels of the key enzyme 1-aminocyclopropane 1-carboxylate synthase 1 (ACS1) of ethylene biosynthesis (Fig. 6). These observations suggest a role for CS in blocking ethylene biosynthesis and preventing woolliness in peach. Moreover, the transcriptional reprogramming of auxin biosynthesis and signaling genes in response to exogenous GA may indicate its involvement in the complex wooly phenotype of cold stored peaches. The extensive transcriptional changes observed in response to GA application (Fig. 2, Additional file 1: Table S1) and the overrepresentation of genes associated to cellular and developmental processes (Table 1, Fig. 3) and hormone metabolism (Fig. 6) in the genome wide transcriptional analyses indicate that reduced incidence of chilling induced damages is likely to result from extensive developmental reprogramming mediated by hormones in response to GA application.

### Effect of CS on peach fruit

Low temperatures have been demonstrated to affect the activity of enzymes associated to pectin metabolism in fruit [2], although the magnitude and direction of the reported effects are largely discrepant [5, 11, 29–32]. In contrast to its biochemical effect, genome wide transcriptional analyses have demonstrated a preferential repressive function of low temperatures on gene expression in peach (Figs. 2, 4, 5 and 6). Low temperatures were also able to promote the transcription of a small number of genes associated to cell wall metabolism, and those related to carbohydrate metabolism and endomembrane transport (Fig. 4). Interestingly, the repressive effect of cold on gene expression was completely or partially alleviated by the exogenous application of GA for sequences associated to hormone metabolism and signal transduction, such as gibberellin biosynthesis oxidases and ethylene responsive transcription factors (AP2/ERF) (Fig. 6). Low temperatures had a significant repressive effect on the transcription of GA signaling components, although exogenous hormone application released the repression of *GID*1 and *SPT* homologs (Fig. 6).

In plants, the conserved C-repeat binding factor (CBF) pathway has been associated to low temperature tolerance in a wide range of evolutionary distinct species [33]. The CBF signaling network is positively regulated by the circadian clock components CCA1 and LHY [34] and functions downstream of the INDUCER OF CBF EXPRESSION 1 (ICE1) protein [33]. The hormone salicylic acid (SA) has also been shown to participate in low temperature responses in plants [35]. In peach, several genes exhibiting similarity to known partners of the CBF and SA pathway were transcriptionally affected by low temperatures (Fig. 6).

Our genome wide transcription profiling has demonstrated the antagonistic role of GA on low temperature transcriptional repression in fruit (Figs. 4, 5 and 6). These findings may be associated to the distinct functional roles of gibberellins throughout plant development, since the hormone is associated to stress tolerance during germination and seedling establishment [36] and is involved in stress sensitivity in determined vegetative tissues [37].

### The role of cell wall metabolism and hormone interplay in woolliness

Woolliness is a complex phenotype observed in fruit after CS, consisting in a severe loss of juice and dry texture of the fruit flesh [2]. Peaches (*Prunus persica* Batsch.) and nectarines (*P. persica* var. *nectarina* Ait.) are highly subjected to the physiological disorder [2, 3, 8], although some cultivars exhibit reduced woolliness incidence by exogenous GA application previous to pit hardening [5, 6]. The reduction of polygalacturonase (PG) activity, and subsequent reduction in water-soluble pectins and increase in sodium-carbonate-soluble pectins, by low temperatures is considered the biochemical basis for woolliness in peaches [2]. Employing genome wide and time course expression analyses we have demonstrated that extensive transcriptional regulation occurs in stored peaches and may contribute to the biochemical changes that appear to underlie woolliness. Consistent with the reduced enzymatic activity, CS was demonstrated to preferentially repress gene expression in peach (Figs. 2, 4, 5, 6 and 7). Approximately, half of the peach transcriptome exhibited significant differential expression patterns in response to GA and CS, suggesting that extensive genetic reprogramming is the basis for the known biochemical changes during ripening.

The reduction in woolliness incidence by preharvest GA application to immature fruit, at early endocarp hardening stage is consistent with changes in the hormone mediated developmental transitions controlling ripening. In peach, low temperatures repressed the transcription of a GA receptor homolog *GID*1, a DELLA receptor coding sequence and an *ALC*/*SPT* transcriptional regulator gene. In contrast, exogenous application of the hormone up regulated these genes. In GA treated fruits, chilling for 15 days restored the expression levels of *GID*1 and *ALC*/*SPT*, whereas the combination of GA and CS further decreased the transcription of the DELLA coding sequence. In *Arabidopsis*, there is no evidence of temperature regulation of the transcription of the partially redundant *ALC* and *SPT* [23, 38, 39]. However, other members of the bHLH family of ALC and SPT, the PHYTOCHROME-INTERACTING FACTORS (PIFs), have been demonstrated to be involved in a wide range of temperature controlled processes in *Arabidopsis* [40–42].

Biochemical changes and modifications in enzyme activity are considered the most important mechanism responsible for chilling induced damage in peaches [2, 4, 8]. Our genome wide expression analyses suggest that transcriptional regulation is likely to contribute to the biochemical changes associated to postharvest processes in peach. Moreover, the over representation of genes associated to developmental ontology classes and the large number of hormone metabolism differentially regulated in response to CS and GA in peaches indicate that the physiological disorder and its reduced incidence in response to the hormone treatment are subjected to developmental regulation, as shown for other species [43–45]. In addition, genes related to photosystem I and II are differentially expressed in woolly fruit. These findings may contribute to explain the changes in chlorophyll a fluorescence during cold storage [46]. Transcript accumulation of *PSBY* is modified before chilling injury (after 15 days). Thus, the expression profile of the gene can be used as a wooliness incidence marker.

## Conclusion

In the current work, we have investigated the factors underlying the prevention of chilling injuries in peaches by the application of GA at the initial stages of pit hardening, coupling physiological analyses of a responsive cultivar to global transcriptional profiling. We have confirmed the involvement of cell wall, hormone and stress metabolism in controlling the fruits responses to low temperatures during storage and have demonstrated that GA application at the early stages of endocarp hardening alone or coupled with cold storage trigger complex transcriptional reprogramming in peaches. Our data demonstrated the transcriptional control of GA receptor and signaling partner ALC/SPT in response to the hormone application and cold storage, suggesting GA controlled developmental processes, such as determination of endocarp borders, may be involved in the determination of chilling injury susceptibility in peach.

## Methods

### Plant material

Peach [*P. persica* (L.) Batsch cv. Chimarrita] fruits used in the current study were obtained from a commercial orchard of ‘Chimarrita’ clones grafted on ‘Capdeboscq’. Permission to sample and harvest the fruits was requested before the experiments and granted by the orchard owner. ‘Chimarrita’ fruits have white, melting flesh that is semi-adherent to the endocarp, The ripening cycle is intermediate in Brazilian growth conditions, lasting 115 days from anthesis to fruit ripening. Three biological replicates, each consisting of 20 trees, were selected based on uniformity and conducted as follows: for GA treatment, plants were sprayed at 400 L ha^−1^ of a solution of 50 mgL^−1^ of GA (Pro-Gibb, Abbot Laboratories, North Chicago, USA, 10 % m/m) supplemented with 0.05 % (v/v) surfactant (Silwet, Momentive Performance Materials Inc., Waterford, NY) pH 4.5, at the initial stage of the pit hardening (45 days after anthesis, DAA) (Additional file 3: Figure S2). Untreated control plants were sprayed with the solution without hormone. Fruits from treated and control plants were harvested at light green ground coloration, corresponding to the pre-climacteric stage [6]. Each biological replicate consisted of 280 fruits (14 fruits from each tree), totalizing 840 fruits per treatment.

### Postharvest conditions

Woolliness symptoms are distinguishable in peaches after two days at room temperature (RT, 25 ± 2 °C). Thus, analyses were performed after the 2-day RT period for all sampling points. Fruits were collected at harvest and up to 30 days under CS at 0 ± 0.5 °C and 90 ± 5 % of relative humidity, at five day intervals. Experiments were conducted in triplicates, consisting of 40 fruits. Experimental design and sampling points are schematically represented in Additional file 3: Figure S2.

### Woolliness evaluation

Incidence of woolliness was evaluated by manually squeezing the fruits, as described by Pegoraro et al. [6]. Fruits failing to release juice when squeezed were considered woolly. Normal distribution, homoscedasticity and residue independence of woolliness data were tested by Shapiro Wilk, Hartley test and graphic analyses, respectively. Data were subjected to F test (p ≤ 0.05) ANOVA. Statistically significant results were compared by *t* test (p ≤ 0.05) for gibberellic acid and factor interaction effects, when present. Least significant differences (LSD) between the means were plotted and considered significant in the absence of overlapping vertical bars. Time course effects were investigated by non-linear regression models (p ≤ 0.05), as follows: y = y*0* + ae^bx^, where: y = response of interest variable; y*0* = minimal woolliness; a = maximum estimated value for the response variable; b = slope; x = time (days); e = constant.

### RNA extraction

Samplings for genome wide transcriptional profiling were carried out at harvest and 15 days after CS (Additional file 3: Figure S2). This time point corresponds to the beginning of woolliness occurrence in untreated peaches. Total RNA extraction was carried out from 2 g of pulp, using a cetyltrimethylammonium bromide (CTAB) method described by Zeng Y, Yang [47]. Quantity and quality of the isolated RNA were spectrophotometrically analyzed with NanoDrop (NanoDrop, Thermo Scientific, Wilmington, DE), agarose gel and capillary electrophoresis using 2100 Bioanalyzer (Agilent Technologies, Santa Clara, CA). Hybridizations and qRT-PCR experiments were performed with samples with RIN ranging from 8.5 to 9.3 [48]. Time course expression analyses during CS employed RNA extracted from 100 mg of fruit pulp using PureLink kit (LifeTechnologies, Carlsbad, CA), as described by the manufacturer. Nucleic acid quality and quantity were determined as described previously. All samples were treated with DNase I (LifeTechnologies, Carlsbad, CA) for complete removal of genomic DNA, as confirmed by qPCR employing primers for *TRANSLATION ELONGATION FACTOR* 2 (*TEF2*) gene.

### Microarray hybridization

Microarray hybridizations were performed for biological triplicates of each experimental treatment using the μPEACH3.0 platform [49], with 1000 ng of total RNA for cDNA synthesis, following the protocol described by the manufacturer (Agilent Technologies, Santa Clara, CA). Slides were scanned using the Agilent Scanner and fluorescence data was determined by Agilent Scan Control software following the manufacturer’s instructions. Data were deposited in the Gene Expression Omnibus (GEO) database under accession number GSE71470.

### Microarray data analyses

Microarray data produced by the Agilent microarray scanner were preprocessed by removal of spots considered ‘well above background’ by the Agilent pre-processing software (Agilent Feature Extraction Software). For each probe, intensity data of the two spots were averaged and quantile-normalized using R library ‘preprocessCore’ [50]. The quality of normalized data was assessed employing the LIMMA package [51] in R statistical programming language [51] at Bioconductor, by raw intensity box plots and density plots. Pairwise treatment conditions were compared by MA plots, where M = log_2_array^i^/array^j^ and A = 1/2*log_2_(array^i^*array^j^). Differential gene expression was analyzed using the LIMMA [51] and Gene Set Enrichment Analysis (GSEA) packages [52] at the Multi Experiment Viewer (MeV), EASE Expression Analysis Systematic Explorer version 4.9 software [53], employing a 2 × 2 factorial design on fruits at harvest and submitted to cold storage, with the factors being GA treatment (absent and present) and Cold Storage (present and absent).

The logical relations among the differentially expressed genes in the treatments were identified using the Venny software [54]. Differentially expressed genes were classified by ontology using singular enrichment analysis (SEA) and parametric analysis of gene set enrichment (PAGE) at AgriGo (http://bioinfo.cau.edu.cn/agriGO/index.php) and classified to metabolic pathways using MapMan v. 3.6 ORC1 software [55]. Relevance networks were constructed by comprehensively comparing all features with each other in a pair-wise manner over the treatments, using ȓ ^2^ [56].

### Reverse transcription quantitative PCR (RT-qPCR) expression analyses

Time course analyses of gene expression during CS and microarray profile validations were performed by RT-qPCR. Synthesis of cDNA was carried out from 1000 ng of total RNA using Oligo d(T) (LifeTechnologies, Carlsbad, CA) primers and SuperScriptIII/RNAse Out Mix (LifeTechnologies, Carlsbad, CA), according to the manufacturer recommendations. Primers were designed for coding sequences of the candidate reference genes from the *Prunus persica* genome, available at the Genome Database for Rosaceae (GDR, − http://www.rosaceae.org/), using the default parameters of the software Primer3Plus [57]. Primer data are presented in Additional file 4: Table S2.

Primers were validated by amplification curve analyses employing a pool of cDNAs from all tested conditions, at five distinct concentrations. Oligonucleotide specificity and absence of primer dimers were checked by posttranscriptional dissociation curves. Quantitative PCR was carried at a StepOne^TM^ Real Time PCR System (LifeTechnologies, Carlsbad, CA) and the SYBR^TM^ Green PCR Master Mix (LifeTechnologies, Carlsbad, CA). The reactions started with a denaturation step at 95 °C for 10 min, followed by 40 cycles consisting of 15 s at 95 °C and 1 min at 60 °C, finalized by the dissociation curve with denaturation at 95 °C for 15 s, cooling at 60 °C for 1 min and gradual heating, at 0.3 °C steps, up to 95 °C. A negative, no template control (NTC), was used to confirm the absence of genomic DNA. Expression data were normalized employing *RNA POLYMERASE* II (*RP* II), *UBIQUITIN* C (*UB*C) and *TEF*2 as reference genes and calibrated using samples from fruits kept at RT for two days after harvest, which are considered optimum for human consumption.

### Availability of supporting data

The data set supporting the results of this article is included within the article and its additional files. Microarray hybridization data were deposited in the Gene Expression Omnibus (GEO) database under accession number GSE71460.

## Additional files

Additional file 1: Table S1. Genes exhibiting significant differential expression in peach. Analyses were performed as a factorial 2 × 2 design, using the LIMMA (Linear Models for Microarray and RNAseq Data) package (Smyth, 2005) at Bioconductor. (XLSX 4527 kb) Additional file 2: Figure S1. Expression validation of microarray genes by RT-qPCR for cold stored and GA treated fruits. Represented genes are functionally classified to cell wall metabolism (EXP - ppa014051m, PME - ppa005976m and PG - ppa025787m), photosynthesis light reactions (PSBY - ppa011725m, PSI-L - ppa011229m and PSI-RC - ppa010953m) and redox metabolism (APX - ppa010426m, SOD - ppa009729m and GPX - ppa011681m). Values correspond to the mean ± SD (*n* = 3). (PDF 127 kb) Additional file 3: Figure S2. Schematic representation of the fruit sampling points used in the current study. Sampling of the fruits for molecular and sensory analyses started after two days at room temperature (RT). Triangles represent sampling points for wooliness detection (black), microarray hybridizations (white) and time course expression analyses by RT-qPCR (spotted). (PDF 118 kb) Additional file 4: Table S2. Peach (*Prunus persica* (L.) Batsch.) genome and amplification information on the RT-qPCR primers used in the current study. (PDF 73 kb)

## Abbreviations

ACS: 1-aminocyclopropane 1-carboxylate synthase 1
ALC: ALCATRAZ
ANOVA: analysis of variance
AP2/ERF: APETALA2/ethylene responsive factor
bHLH: basic helix-loop-helix
CBF: C-repeat binding factor
CCA1: circadian clock associated 1
cDNA: complementary DNA
CS: cold storage
CSGA: cold storage_ gibberellic acid
CTAB: cetyltrimethylammonium bromide
DAA: days after anthesis
DELLA: DELLA protein
DNA: deoxyribonucleic acid
EASE: expression analysis systematic explorer
EXP: expansin
GA: gibberellic acid
GAOX1: gibberellin oxidase1
GDR: genome database for rosaceae
GID1: gibberellin insensitive dwarf1
GO: gene ontology
GSEA: gene set enrichment analyses
ICE1: inducer of CBF expression
IND: indehiscent
LHY: late elongated hypocotyl
LIMMA: linear models for microarray data
LSD: least significant differences
MeV: multi experiment viewer
NTC: no template control
PAGE: parametric analysis of gene set enrichment
PG: polygalacturonase
PIFs: phytochrome-interacting factors
PME: pectin methyl esterase
PSBY: photosystem II core complex protein
RNA: ribonucleic acid
RP II: RNA polymerase II
RT: room temperature
RT-qPCR: reverse transcription-quantitative polymerase chain reaction
SA: salicylic acid
SEA: singular enrichment analysis
SPT: SPATULA
TEF2: TRANSLATION ELONGATION FACTOR 2
UBC: UBIQUITIN C
