# Supplementary material for: Transcriptional regulatory networks controlling woolliness in peach in response to preharvest gibberellin application and cold storage
Source: BMC Plant Biol. 2015 Nov 18;15:279. doi: 10.1186/s12870-015-0659-2 (PMC4652400; doi:10.1186/s12870-015-0659-2)
Supplement: Additional file 2: Figure S1. — Expression validation of microarray genes by RT-qPCR for cold stored and GA treated fruits. Represented genes are functionally classified to cell wall metabolism (EXP - ppa014051m, PME - ppa005976m and PG - ppa025787m), photosynthesis light reactions (PSBY - ppa011725m, PSI-L - ppa011229m and PSI-RC - ppa010953m) and redox metabolism (APX - ppa010426m, SOD - ppa009729m and GPX - ppa011681m). Values correspond to the mean ± SD (n = 3). (PDF 127 kb) [file 12870_2015_659_MOESM2_ESM.pdf]

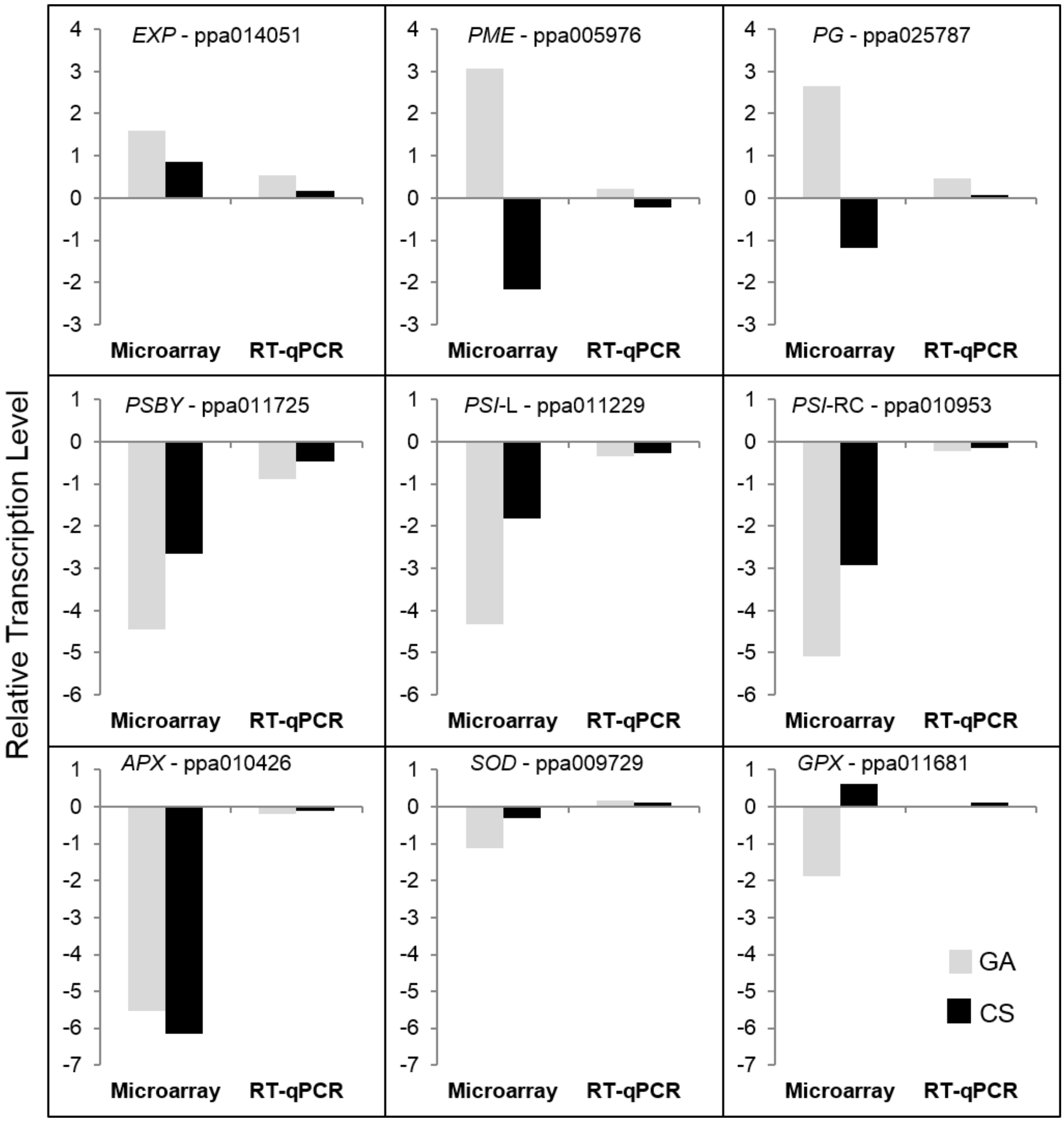

Additional file 2: Figure S1. Expression validation of microarray genes by RT-qPCR for cold stored and GA treated fruits. Represented genes are functionally classified to cell wall metabolism (*EXP* - ppa014051m, *PME* - ppa005976m and *PG* - ppa025787m), photosynthesis light reactions (*PSBY* - ppa011725m, *PSI*-L - ppa011229m and *PSI*-RC - ppa010953m) and redox metabolism (*APX* - ppa010426m, *SOD* - ppa009729m and *GPX* - ppa011681m). Values correspond to the mean ± SD (n = 3).
