# Supplementary material for: Transcriptional regulatory networks controlling woolliness in peach in response to preharvest gibberellin application and cold storage
Source: BMC Plant Biol. 2015 Nov 18;15:279. doi: 10.1186/s12870-015-0659-2 (PMC4652400; doi:10.1186/s12870-015-0659-2)
Supplement: Additional file 3: Figure S2. — Schematic representation of the fruit sampling points used in the current study. Sampling of the fruits for molecular and sensory analyses started after two days at room temperature (RT). Triangles represent sampling points for wooliness detection (black), microarray hybridizations (white) and time course expression analyses by RT-qPCR (spotted). (PDF 118 kb) [file 12870_2015_659_MOESM3_ESM.pdf]

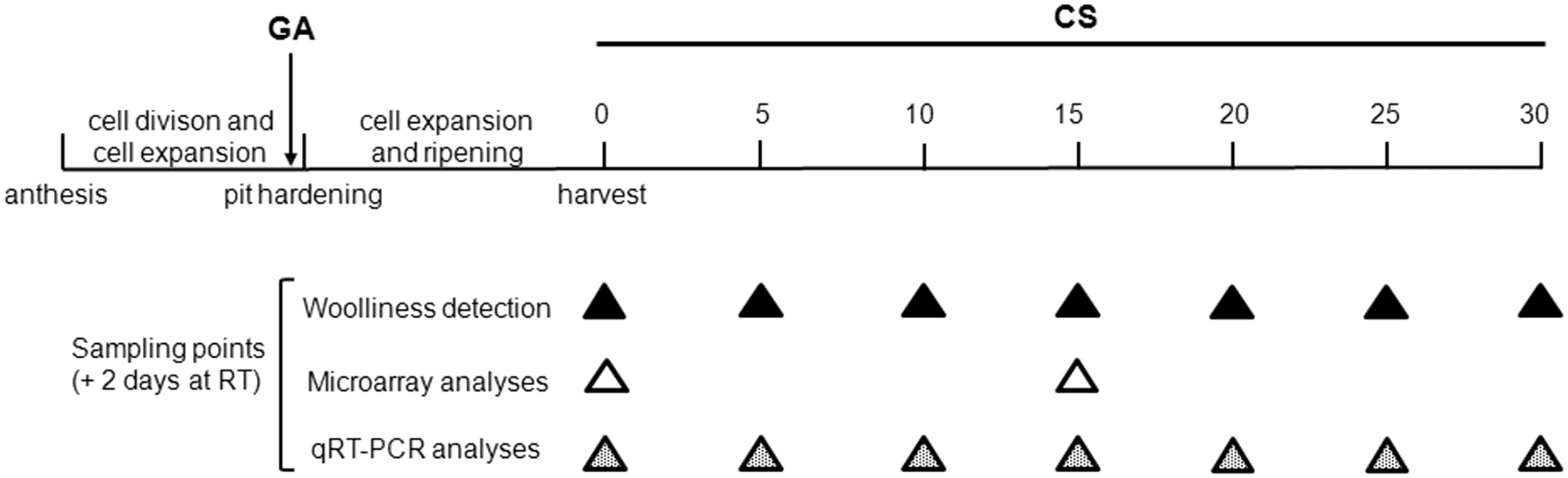

Additional file 3: Figure S2. Schematic representation of the fruit sampling points used in the current study. Sampling of the fruits for molecular and sensory analyses started after two days at room temperature (RT). Triangles represent sampling points for wooliness detection (black), microarray hybridizations (white) and time course expression analyses by RT-qPCR (spotted).
