# Supplementary material for: Transcriptional regulatory networks controlling woolliness in peach in response to preharvest gibberellin application and cold storage
Source: BMC Plant Biol. 2015 Nov 18;15:279. doi: 10.1186/s12870-015-0659-2 (PMC4652400; doi:10.1186/s12870-015-0659-2)
Supplement: Additional file 4: Table S2. — Peach (Prunus persica (L.) Batsch.) genome and amplification information on the RT-qPCR primers used in the current study. (PDF 73 kb) [file 12870_2015_659_MOESM4_ESM.pdf]

Additional file 4: Table S2. Peach (*Prunus persica* (L.) Batsch.) genome and amplification information on the RT-qPCR primers used in the current study.

Gene description/ acronym	Accession code	Primer Sequence (5´to 3´)		Amplicon size (bp)	Temperature of melting (°C)	
		Forward	Reverse		Forward	Reverse
Expansin (*EXP*)	ppa014051m	TGGTGAGTGTTTCTGCT TGC	CAGAGCCTCCGTAGAAG GTG	129	60.03	60.01
Pectin methylesterase (*PME*)	ppa005976m	ACCCACAACACCCAAAA GAA	ATGAGGTTCGGGTGAGT CAG	131	60.25	60.11
Polygalacturonase (*PG*)	ppa025787m	CACCAAAGCCTTCCTCT CTG	GAAGGTGATGGCGTTGT TCT	136	59.98	60.12
Photosystem II core complex protein (*PSBY*)	ppa011725m	AAGTGCATGAGCACCAA CAC	TGTTGATGCTGGGAATTT CA	130	59.76	60.05
Photosystem I subunit L (*PSI* – L)	ppa011229	CCATCAATGGTGATCCC TTC	CCACCTCAATACCCCTGA GA	132	60.13	59.92

Photosystem I reaction center subunit III (*PSI* – RC)	ppa010953	CGTGAGAAGCAGCAGATCAA	AACCCTGCTTGCCATAGTTG	133	60.52	59.98
Ascorbate peroxidase (*APX*)	ppa010426m	AATGCTACCCGACTGTGAGC	TCGGAGCTTTCTCTTGCATT	65	60.3	60.1
Superoxide dismutase (*SOD*)	ppa009729m	TGAGCTTGTGGATGACCTTG	TGCATTGCCAGTGCTTAGAC	61	59.8	60.0
Glutathione peroxidase (*GPX*)	ppa011681m	TATGGCTGAAGGAAGTTCCAAA	TTAAACCACATTGGGAAGCAAC	131	60.9	61.1
Translation elongation factor 2 (*TEF2*)	ppa001368m	GGTGTGACGATGAAGAGTGATG	TGAAGGAGAGGGAAGGTGAAAG	129	59	60
RNA polymerase II (*RP*II)	ppa016873m	TGAAGCATACACCTATGATGATGAAG	CTTTGACAGCACCAGTAGATTCC	128	60	58
Ubiquitin C (*UBC*)	ppa007117m	AAGGCTAAGATCCAAGACAAAGAG	CCACGAAGACGAAGCACTAAG	146	58	58
